# Digital Technologies in Postsurgical Orthopedic Rehabilitation: Scoping Review Mapping Interventions to the CAMS Framework (2020‐2025)

**DOI:** 10.2196/100548

**Published:** 2026-10-20

**Authors:** Ngan Kuen Lai, Hai Huang, Tet Khuan Chen, Muhammad Nur Affendy Nor'a, Jeffery Sijore, Wei Yang Chen, YongSheng Luo

**Affiliations:** 1 School of Computing, Asia Pacific University of Technology and Innovation (APU), Jalan Teknologi 5, Taman Teknologi Malaysia, Wilayah Persekutuan Kuala Lumpur, 57000, Malaysia, 60 8996 1000; 2 School of Data Science, Zhuhai College of Science and Technology, Zhuhai, China

**Keywords:** telerehabilitation, orthopedic surgery, CAMS framework, virtual reality, AI, digital rehabilitation, Coordination, Alignment, Motivation, and Strength framework

## Abstract

**Background:**

Digital technologies have become common in postoperative orthopedic rehabilitation. The tendency toward this adoption appeared in the past decade. However, no standard method exists to classify their functional capabilities. This absence hinders practical implementation. Clinicians currently lack a structured framework to distinguish between the functional capabilities of different digital rehabilitation technologies effectively.

**Objective:**

This scoping review aims to map digital interventions used in postsurgical orthopedic rehabilitation to a proposed Coordination, Alignment, Motivation, and Strength (CAMS) framework and to identify research trends, functional coverage, and gaps in current evidence.

**Methods:**

A systematic search was conducted across 5 databases: MEDLINE (via PubMed), Scopus, IEEE Xplore, Cochrane Central Register of Controlled Trials (CENTRAL), and the Physiotherapy Evidence Database (PEDro), following PRISMA-ScR (Preferred Reporting Items for Systematic Reviews and Meta-Analyses extension for Scoping Reviews) guidelines. Studies were mapped to the proposed CAMS framework pillars. Multiple validated appraisal tools were applied descriptively according to study design: version 2 of the Cochrane risk-of-bias tool for randomized trials (RoB 2; n=32), the Newcastle-Ottawa Scale (NOS) for observational studies (n=2), and the Methodological Index for Non-Randomized Studies (MINORS) for nonrandomized designs (n=1).

**Results:**

A total of 35 studies met the inclusion criteria. Coordination was the most frequently addressed pillar (29/35, 83%), followed by Motivation (26/35, 74%). Alignment was addressed in 37% (13/35) of studies, while Strength was the least represented pillar (12/35, 34%). Overall, 49% (17/35) of studies were rated “low risk” overall on the primary appraisal tool, 46% (16/35) demonstrated “some concerns,” and 6% (2/35) were rated “high risk,” with performance bias identified as a consistent methodological characteristic across the corpus. Delivery models were predominantly hybrid (14/35, 40%) or home-based (13/35, 37%), with 8 studies (8/35, 23%) using an exclusively clinical model.

**Conclusions:**

Digital health has shown a notable increase in the kinematic monitoring feature, offering effective solutions to movement quality (Coordination and Alignment) and psychological engagement (Motivation). Nevertheless, there is a consistent gap in strength-related studies, as current decentralized tools lack the capability to quantify physiological load. The next phase of innovations should be a shift to integrated 4-pillar solutions that would coordinate resistance-based training with high-precision biomechanical monitoring. The CAMS framework is proposed as an exploratory classification tool to guide future research design and technology evaluation in postsurgical digital rehabilitation.

## Introduction

### The Paradigm Shift Toward Decentralized Care

Traditionally, musculoskeletal rehabilitation has been localized in traditional ambulatory clinics, where it is hand-supervised and facilitated by a clinician. Nevertheless, the increasing clinical expenses, geographic access limitations, and the need for customized care have dictated a shift toward decentralized telerehabilitation. Digital health interventions have ceased to be alternative solutions, but they have already become a part of the continuum of care, helping to transform hospital-based acute recovery into independent, home-based maintenance [1]. Although this type of therapy has been rapidly adopted, there is still no standard approach to assessing the functional breadth of these tools.

### Immersive Technologies: The Coordination-Motivation Axis

Gamification has transformed the engagement of patients by integrating virtual reality (VR) and augmented reality (AR). Immersive VR environments have been associated with reduced pain perception and perceived exertion during physical activity in the included literature [2], though this review’s descriptive design does not establish a causal or clinical analgesic effect. AR systems, on the contrary, improve the rehabilitation experience through superimposing digital instructions directly onto the physical surroundings of the patient [3]. These technologies guide users through accurate movement paths by giving spatial-sensitive and real-time instructions. The tools are particularly efficient in the Coordination and Motivation pillars of the Coordination, Alignment, Motivation, and Strength (CAMS) structure, providing instant visual feedback required when learning a neuromuscular motor skill [4].

### Markerless AI and Computer Vision: Ensuring Alignment

The invention of the markerless pose estimation using cameras has transformed alignment tracking. Contrary to wearable sensors, whose use can be affected by sensor drift or even being moved around during intense activity, AI-based computer vision measures the angles of joints and postural balance through normal smartphone cameras [5]. The technology plays a vital role in detecting compensatory movements, that is, patients use secondary muscular groups to avoid pain or weakness, thus providing the clinical quality of the rehabilitation setting in home-based unsupervised environments [6].

### Robotics and the “Strength Gap”

The Strength pillar uses robotics and haptics to monitor movement quality via computer vision. Assist-as-needed strategies are used by the robotic devices to facilitate adaptive plasticity and functional recovery [7]. The high price and mechanical nature of these tools, however, tend to limit their application to the clinical environment and introduce a major technological gap in the realm of home-based strength training [8].

### The CAMS Framework: A Proposed Taxonomy

Although there are numerous digital tools that provide generic activity tracking, they tend to overlook the subtle issues of clinical recovery. The CAMS framework is proposed in this review as an exploratory conceptual taxonomy to classify digital rehabilitation technologies according to the primary functional domains they address. The framework is not presented as a validated or standardized clinical tool; rather, it serves as a structured lens to support evidence mapping and gap identification. The classification of interventions based on these pillars helps us to understand whether the device just measures the quantity (eg, the number of repetitions) or the quality of recovery by means of biomechanical alignment and neuromuscular coordination. The framework positions motivation as the primary driver for adherence, ensuring that digital movement is viewed as a holistic clinical event rather than an isolated mechanical task. The rationale for selecting these 4 pillars reflects the authors’ analysis of the digital rehabilitation literature: CAMS represent the functional domains most commonly and directly targetable by current digital health technologies. Other clinically relevant domains, including pain, range of motion, balance, gait function, and return to sport, are treated as downstream composite outcomes that emerge from the interplay of these 4 primary domains, rather than being directly addressed by a single technology class. This framing is the authors’ conceptual contribution and is subject to empirical validation.

## Methods

### Study Design and Protocol Registration

This scoping review aimed to address the following question:

1. What types of digital technologies are used in postsurgical orthopedic rehabilitation?
2. How can they be classified across functional domains using the CAMS framework?
3. What research gaps exist in the functional coverage of current digital rehabilitation tools?

This study was conducted as a scoping review following the PRISMA-ScR (Preferred Reporting Items for Systematic Reviews and Meta-Analyses Extension for Scoping Reviews) guidelines [9]. A scoping review methodology was selected due to the heterogeneity of intervention types, outcome measures, and study designs, and the aim to map the breadth and characteristics of existing digital rehabilitation technologies rather than to evaluate their clinical effectiveness or synthesize outcome data. The protocol in the review was prospectively registered in the International Prospective Register of Systematic Reviews (PROSPERO) with a registration number of CRD420251062131.

### Search Strategy and Information Sources

A comprehensive systematic search was conducted across 5 electronic databases, namely MEDLINE (via PubMed), Scopus, IEEE Xplore, Cochrane Central Register of Controlled Trials (CENTRAL) and Physiotherapy Evidence Database (PEDro). Web of Science was not accessible through the authors’ institutional subscriptions and was therefore not included in the search; this is acknowledged as a limitation.

The initial systematic search, conducted on December 15, 2025, identified 1078 records across the 5 databases, supplemented by 55 additional records identified through bidirectional citation tracking (Google Scholar snowballing), yielding 1133 records in total. Duplicate records were identified and removed using EndNote (Clarivate), supplemented by manual verification, reducing the total to an estimated 818 records for title and abstract screening. Searches covered literature published between January 2020 and December 2025, restricted to English-language publications. The search strategy used MeSH and free-text terms related to digital rehabilitation, orthopedic surgery, and postoperative care. The complete search strings for all 5 databases were as follows.

1. MEDLINE (via PubMed): (“telerehabilitation”[MeSH Terms] OR “Telemedicine”[MeSH Terms] OR “Mobile Applications”[MeSH Terms] OR “virtual reality”[MeSH Terms] OR “virtual rehabilitation”[tiab] OR “wearable electronic devices”[MeSH Terms] OR “augmented reality”[tiab] OR “mHealth”[tiab] OR “mobile health”[tiab] OR “digital rehabilitation”[tiab] OR “digital health”[tiab] OR “smartphone”[tiab]) AND (“orthopedic procedures”[MeSH Terms] OR “Orthopedics”[MeSH Terms] OR “arthroplasty, replacement, knee”[MeSH Terms] OR “arthroplasty, replacement, hip”[MeSH Terms] OR “anterior cruciate ligament reconstruction”[MeSH Terms] OR “rotator cuff”[MeSH Terms] OR “orthopedic rehabilitation”[tiab] OR “post-surgical rehabilitation”[tiab] OR “orthopedic surgery”[tiab]) AND (“postoperative care”[MeSH Terms] OR “rehabilitation”[MeSH Terms] OR “recovery of function”[MeSH Terms]). Filters: 2020‐2025, English language, Human subjects.
2. Scopus: TITLE-ABS-KEY((“telerehabilitation” OR “mobile health” OR “virtual reality” OR “wearable electronic device*” OR “augmented reality” OR “mHealth” OR “digital rehabilitation”) AND (“orthopedic procedure*” OR “knee arthroplasty” OR “hip arthroplasty” OR “anterior cruciate ligament reconstruction” OR “rotator cuff” OR “orthopedic rehabilitation” OR “post-surgical rehabilitation”) AND (“postoperative care” OR “rehabilitation” OR “recovery of function”)) AND PUBYEAR>2019 AND PUBYEAR<2026 AND LANGUAGE(english).
3. IEEE Xplore: (“telerehabilitation” OR “mobile health” OR “virtual reality” OR “wearable device” OR “augmented reality” OR “mHealth” OR “digital rehabilitation”) AND (“orthopedic” OR “knee arthroplasty” OR “hip arthroplasty” OR “ACL reconstruction” OR “rotator cuff” OR “orthopedic rehabilitation”) AND (“postoperative” OR “rehabilitation” OR “recovery of function”), limited to 2020‐2025.
4. Cochrane CENTRAL: #1 telerehabilitation OR “mobile health” OR “virtual reality” OR “wearable electronic devices” OR “augmented reality” OR mHealth OR “digital rehabilitation”; #2 “orthopedic procedures” OR “knee arthroplasty” OR “hip arthroplasty” OR “anterior cruciate ligament reconstruction” OR “rotator cuff” OR “orthopedic rehabilitation”; #3 “postoperative care” OR rehabilitation OR “recovery of function”; #1 AND #2 AND #3, limited to 2020‐2025.
5. PEDro: Advanced search conducted using the following filters: Body part=knee, hip, shoulder; Subdiscipline=orthopedics; Method=clinical trial; combined with free-text terms “telerehabilitation,” “digital rehabilitation,” “wearable,” and “virtual reality” in the title or abstract field, limited to 2020‐2025.

The search string provided above represents a reconstruction of the search logic applied, developed to document the conceptual scope of the original search (technology type AND orthopedic procedure AND rehabilitation or recovery outcome) for reproducibility purposes. Minor variation in exact result counts between the original search and this reconstruction may reflect index growth in MEDLINE (via PubMed) since the original search date, as well as evolving MeSH term indexing.

The CAMS framework was not used as a search term, as it is a novel taxonomy proposed in this review; rather, CAMS domain concepts informed post hoc mapping of retrieved studies. Google Scholar was used to perform bidirectional citation tracking (forward and backward snowballing), yielding 55 additional records (40 backward and 15 forward), which are documented as a separate identification source in the PRISMA-ScR flow diagram ().

### Eligibility Criteria

Studies were included based on the population-concept-context (PCC) framework recommended for scoping reviews:

- Population: Adults undergoing postsurgical orthopedic rehabilitation following procedures such as anterior cruciate ligament (ACL) reconstruction, total knee or hip arthroplasty, or rotator cuff repair.
- Concept: Digital health technologies used as primary or adjunct rehabilitation tools, including VR, AR, AI-based computer vision, wearable sensors, or mobile health (mHealth) applications, and their functional classification across the CAMS domains.
- Context: Primary peer-reviewed experimental studies, including randomized controlled trials (RCTs), feasibility RCTs, prospective cohort studies, and nonrandomized controlled trials with an active comparator (which may include usual or standard care), published in English between January 2020 and December 2025, conducted in clinical, hybrid, or home-based settings. The full inclusion and exclusion criteria are presented in Textbox 1.

Studies were excluded if they involved nonsurgical populations, healthy participants, neurorehabilitation (eg, stroke), purely observational designs without an active digital intervention, or secondary research (reviews and meta-analyses). Only peer-reviewed articles published in English were included. A total of 3 studies identified during full-text review were subsequently excluded: 1 published in a nonindexed journal, 1 available only as an unpublished preprint, and 1 identified as a duplicate publication of an already-included trial. These exclusions are documented in the PRISMA-ScR flow diagram.

Textbox 1. **Inclusion and exclusion criteria for a scoping review of digital technologies in postsurgical orthopedic rehabilitation, covering studies published between January 2020 and December 2025. (Population-concept-context framework applied per Preferred Reporting Items for Systematic Reviews and Meta-Analyses extension for Scoping Reviews guidance.)**

**Inclusion Criteria**

- Adults (≥18 years) undergoing postsurgical orthopedic rehabilitation following anterior cruciate ligament (ACL) reconstruction, total knee or hip arthroplasty, or rotator cuff repair
- Active digital health technology as primary or adjunct intervention (virtual reality, augmented reality, AI computer vision, wearable sensors, or mHealth application)
- Peer-reviewed experimental studies: randomized controlled trials (RCTs), feasibility RCTs, prospective cohort studies, or nonrandomized controlled trials with an active comparator (which may include usual or standard care)
- Published in English, January 2020 to December 2025
- Conducted in clinical, hybrid, or home-based settings
- Reported ≥1 functional rehabilitation outcome measurable by the digital intervention

**Exclusion Criteria**

- Nonsurgical or healthy participant populations
- Neurorehabilitation populations (eg, stroke and traumatic brain injury)
- Passive or diagnostic-only technologies without an active rehabilitation component
- Secondary research (systematic reviews, meta-analyses, and narrative reviews)
- Conference abstracts, editorials, or nonpeer-reviewed grey literature
- Unpublished preprints
- Non-English language publications

### Study Selection and Data Charting

Titles and abstracts were independently screened by 2 reviewers (WYC and YSL), followed by full-text assessment of potentially eligible studies. Discrepancies were resolved through discussion and consensus.

Data charting was conducted using a standardized form developed and pilot-tested by the research team prior to full extraction. Charting was performed independently by 2 reviewers (WYC and YSL), with discrepancies resolved through consensus.

The following variables were extracted: study design, patient population, type of digital intervention, rehabilitation phase, rehabilitation setting (clinical, home-based, or hybrid), anatomical focus (upper limb, lower limb, or full body), and reported outcome measures. Interventions were subsequently categorized according to the proposed CAMS framework for structured analysis.

### CAMS Framework Classification

To characterize the clinical focal point of each technology, interventions were mapped to the 4 pillars of the CAMS framework (Figure 1):

- Coordination: focus on neuromuscular control, movement sequencing, and timing.
- Alignment: focus on postural integrity and the mitigation of compensatory movement patterns.
- Motivation: focus on patient engagement, adherence, and psychological involvement.
- Strength: focus on mechanical loading, resistance training, and force production.

**Figure 1.**
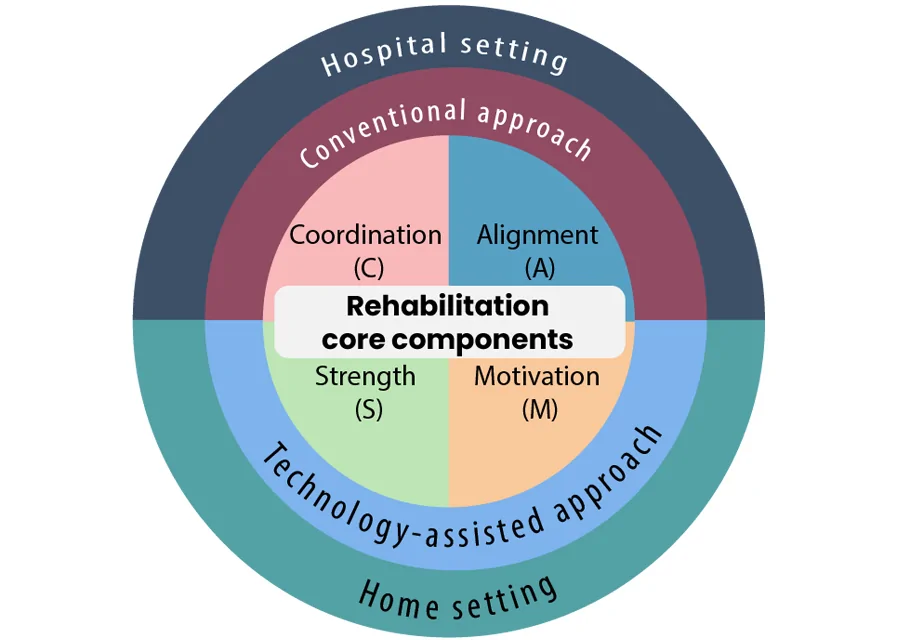
The Coordination-Alignment-Motivation-Strength (CAMS) framework was applied to classify digital rehabilitation technologies in postsurgical orthopedic rehabilitation, based on 35 studies [1,3,10-42] published between 2020 and 2025.

Interventions were permitted to map to multiple CAMS domains where the digital tool addressed more than 1 functional construct (eg, an AR system providing joint angle biofeedback [Alignment] while tracking repetition speed [Coordination]). CAMS domain classification followed the same independent dual-reviewer process described above, using predefined operational coding criteria. Each reviewer (WYC and YSL) independently assigned binary codes (present or absent) to each of the 4 CAMS pillars for every included study. Overall percentage agreement across all pillar-study combinations (n=140 binary decisions: 35 studies × 4 pillars) was 94.3%, with discrepancies resolved through discussion and adjudicated by a third senior reviewer (NKL). Given the high prevalence of the Coordination pillar (29/35, 83%), Cohen kappa was not reported due to the well-documented prevalence-bias paradox [43]. The CAMS framework was applied post hoc after study selection was complete and was not used as an eligibility criterion at any stage of screening or selection. Coding in Table 1 is based on the primary intervention design and reported functional outcomes of each study; an X indicates the digital tool explicitly addressed that CAMS domain as a primary functional target.

The CAMS framework is proposed as an initial conceptual classification model to organize and interpret the functional domains targeted by digital rehabilitation technologies. As a newly proposed framework, CAMS has not yet undergone formal validation and is presented here as an exploratory tool for structured analysis. The CAMS pillars were selected because they represent key modifiable domains commonly targeted by digital rehabilitation technologies. Other clinically relevant outcomes such as pain, balance, and quality of life were intentionally treated as downstream or composite outcomes influenced by these 4 primary domains rather than independent intervention targets. It is not intended as a validated evaluative tool but rather as a structured lens to support synthesis and identify gaps in this research. Future work should focus on empirical validation of the framework across clinical and technological contexts.

### Methodological Quality Appraisal

Methodological quality appraisal is optional in scoping reviews and was conducted here to characterize the methodological profile of the included studies, not as a basis for exclusion. Given the heterogeneity of study designs in the final corpus, multiple appraisal tools were applied according to study design: the Cochrane Risk of Bias 2 (RoB 2) tool was applied to all randomized controlled trials (n=32) [1,3,10-22,24-30,32-38,40-42] including feasibility RCTs across 5 domains (randomization process, deviations from intended interventions, missing outcome data, outcome measurement, and selection of reported results); the Newcastle-Ottawa Scale (NOS) was applied to prospective cohort and retrospective observational studies (n=2) [23,31]; and the Methodological Index for Non-Randomized Studies (MINORS) was applied to quasi-experimental and nonrandomized controlled trials (n=1) [39]. PEDro scale scores were additionally reported to enable comparability with prior physiotherapy rehabilitation literature. Two reviewers (WYC and YSL) independently appraised each study, with discrepancies resolved by the same third senior (NKL) reviewer noted above. Appraisal results are summarized descriptively in Table 2 and were not used to weight or exclude studies.

### Data Synthesis and Analysis

A narrative synthesis was conducted because of a high level of heterogeneity in digital modalities, intervention protocols, and outcome measures. The synthesis of findings was based on a thematic mapping approach that is congruent with the CAMS framework. Descriptive statistics were used to summarize the use of digital tools in terms of setting and anatomical targets. Temporal trends and heatmaps were used to depict how the digital orthopedic environment would evolve and identify some technological gaps in the time frame from 2020 to 2025.

## Results

### Overview

The initial systematic search identified 1078 records across the 5 databases, supplemented by 55 additional records identified through citation tracking, yielding 1133 records in total. The total number of records after the elimination of duplicates was an estimated 818 to be subjected to screening in relation to title and abstract. After this step, an estimated 692 records were eliminated because they failed to meet the main inclusion criteria. Among the estimated 126 reports that had been requested to be retrieved, 5 were unavailable in full text. As a result, an estimated 121 reports were mapped as being eligible.

After a rigorous full-text review, an estimated 86 studies were excluded: 32 involved nonsurgical cohorts, 26 used only diagnostic or passive technologies, 25 did not report any active functional rehabilitation outcome measurable by the digital intervention (eg, range of motion, gait parameters, adherence, strength, or patient-reported functional outcomes), and 3 were excluded following post hoc eligibility verification (1 published in a nonindexed journal; 1 an unpublished preprint; 1 duplicate publication). In total, 35 studies [1,3,10-42] met all inclusion criteria and were included in the final synthesis, representing an inclusion rate of 3.1% (35/1133). This rate is consistent with and higher than comparable reviews in the digital rehabilitation field: a systematic review of digital home rehabilitation for stroke survivors reported 0.13% (13/9873) [44], and a scoping review of AI-driven virtual rehabilitation reported 0.38% (13/3458) [45]. The CAMS framework was applied post hoc after study selection was complete and was not used as an eligibility criterion at any stage of the screening or selection process. The PRISMA-ScR flow diagram (Figure 2) describes the full selection process.

**Figure 2.**
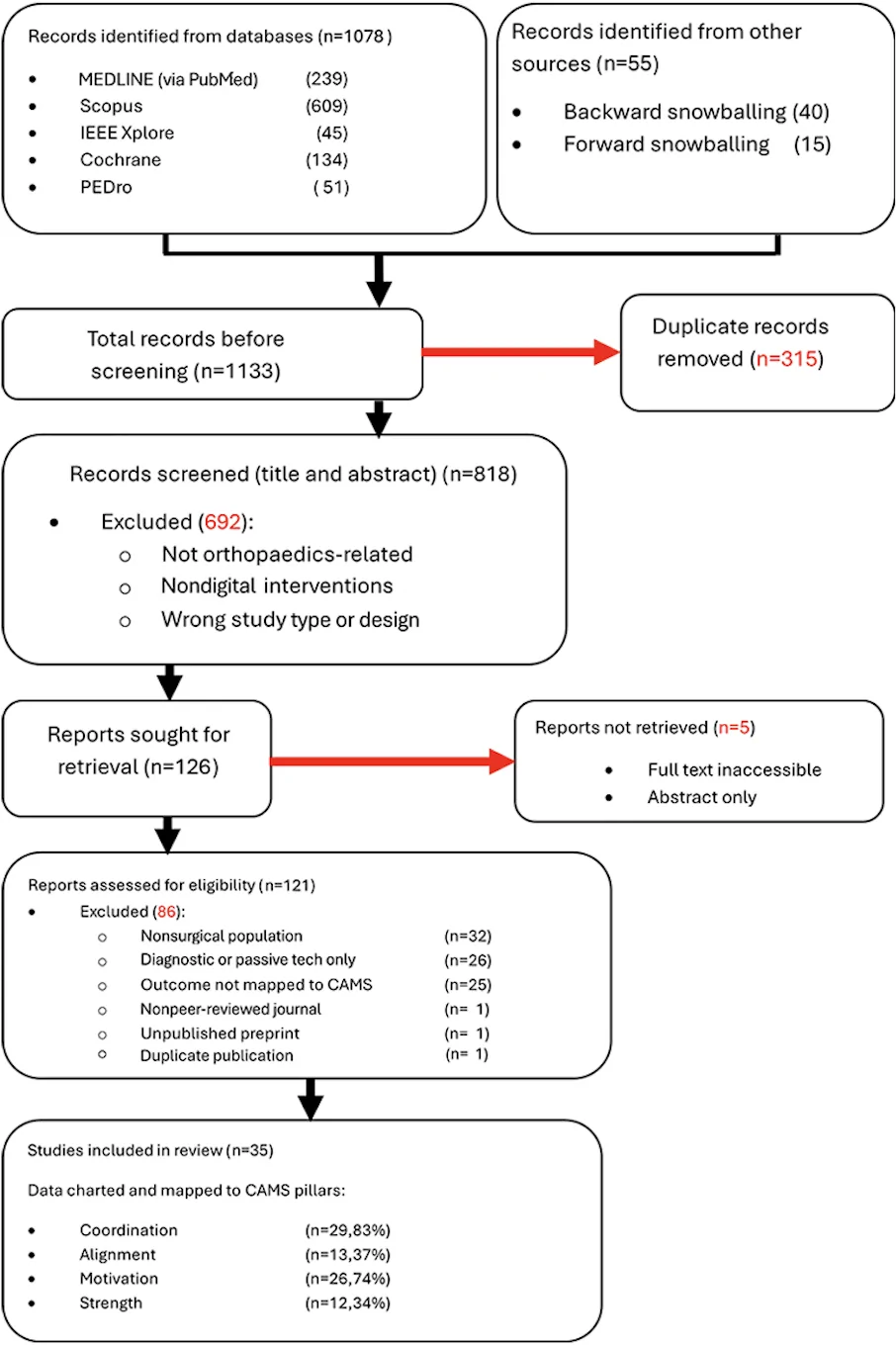
PRISMA-ScR (Preferred Reporting Items for Systematic Reviews and Meta-Analyses extension for Scoping Reviews) flow diagram of study identification, screening, and inclusion for a scoping review of digital technologies in postsurgical orthopedic rehabilitation, 2020‐2025. CAMS: Coordination, Alignment, Motivation, and Strength.

### Study Selection and Characteristics

The reviewed cohort is a pool of evidence that is specialized on the orthopedic postsurgical populations applying active digital rehabilitation interventions. Table 1 represents an overall outline of the studies included and their mapping to the pillars of CAMS.

**Table 1.** Anatomical target, delivery setting, technology type, and CAMS pillar mapping among 35 studies of digital postsurgical orthopedic rehabilitation technologies published between 2020 and 2025. Note: “X” indicates the digital tool explicitly addressed the corresponding CAMS domain as a primary functional target, based on independent dual-reviewer coding (94.3% overall percentage agreement; see Methods). Row numbers (No.) are sequential table identifiers and do not correspond to reference list numbers; full citations for each study are provided in the reference list, numbered in order of first citation in the text.

No.	Year	Ref. no.	Anatomical target	Setting	Technology type	C^a^	A^b^	M^c^	S^d^
1	2025	Chen et al [10]	Lower limb (knee)	Home	Cloud prescription	✓	—^e^	✓	X
2	2025	Dajpratham et al [11]	Lower limb (hip fracture)	Hybrid	Teleresistance	✓	—	✓	✓
3	2025	O’Reilly et al [12]	Upper limb (shoulder)	Hybrid	Telerehabilitation	✓	—	✓	—
4	2025	Sadiq et al [13]	Lower limb (knee)	Hybrid	Web-based+sensor	✓	✓	✓	✓
5	2025	Yuan et al [14]	Lower limb (ACL^f^)	Hybrid	Telerehabilitation	✓	—	✓	✓
6	2025	Zhou et al [15]	Lower limb (hip)	Hybrid	Mobile app	✓	—	✓	—
7	2024	Christiansen et al [16]	Lower limb (knee)	Hybrid	Telehealth+sensor	✓	—	✓	—
8	2024	Lim et al [3]	Lower limb (ACL)	Home	AR^g^	✓	✓	✓	✓
9	2024	Xie et al [17]	Lower limb (knee)	Clinical	Intelligent monitor	✓	✓	—	—
10	2024	Zhao et al [18]	Lower limb (knee)	Home	Smartphone app	—	—	✓	✓
11	2024	Shin et al [19]	Lower limb (knee)	Clinical	Immersive VR^h^	✓	✓	—	—
12	2023	Guo et al [20]	Lower limb (ACL)	Home	mHealth^i^ app	✓	—	✓	—
13	2023	Duong et al [21]	Lower limb (knee)	Hybrid	Digital interventions	✓	—	✓	—
14	2023	Höher et al [22]	Lower limb (knee)	Home	Sensor-based system	—	—	✓	—
15	2023	Lebleu et al [23]	Lower limb (knee)	Hybrid	Digital rehabilitation	✓	—	✓	—
16	2023	Osterloh et al [24]	Lower limb (hip or knee)	Hybrid	Digital group rehab	✓	—	✓	✓
17	2023	Shim et al [25]	Upper limb (shoulder)	Hybrid	Digital health system	✓	✓	✓	—
18	2023	Shim et al [26]	Lower limb (knee)	Hybrid	AR Digital health care	✓	✓	—	✓
19	2023	Gsangya et al [27]	Lower limb (ACL)	Clinical	Immersive VR	✓	✓	—	✓
20	2023	Gür and Başar [28]	Lower limb (knee)	Clinical	VR	—	—	✓	—
21	2023	Nuevo et al [29]	Lower limb (knee)	Hybrid	Telerehab platform (IMU^j^)	✓	—	✓	✓
22	2023	Mojica et al [30]	Lower limb (meniscectomy)	Home	Video telerehab	✓	—	—	—
23	2022	Hong et al [31]	Lower limb (knee)	Home	Digital platform	✓	—	✓	—
24	2022	Marley et al [32]	Upper limb (shoulder)	Home	Gamification or sensors	✓	✓	✓	—
25	2022	Pournajaf et al [33]	Lower limb (knee)	Clinical	VR Serious games	✓	✓	—	—
26	2022	Fuchs et al [34]	Lower limb (knee)	Clinical	VR	—	—	✓	—
27	2022	Zavala-González [35]	Lower limb (hip)	Clinical	VR (Wii balance)	✓	✓	—	—
28	2022	Fascio et al [36]	Lower limb (hip)	Home	Tablet+wearable	✓	—	✓	—
29	2022	Şahi̇n et al [37]	Lower limb (knee)	Home	SMS or call reminders	—	—	✓	—
30	2021	Dunphy et al [38]	Lower limb (ACL)	Hybrid	TRAK-ACL^k^ app	✓	—	✓	—
31	2020	Prvu Bettger et al [1]	Lower limb (knee)	Hybrid	Virtual therapy	✓	✓	✓	—
32	2020	Winjen et al [39]	Lower limb (hip)	Home	Tablet app or coaching	✓	—	✓	—
33	2020	Bell et al [40]	Lower limb (knee)	Home	Wearable motion tracking	✓	—	—	—
34	2020	Gianola et al [41]	Lower limb (knee)	Clinical	VR (inpatient)	—	✓	—	✓
35	2020	Nelson et al [42]	Lower limb (hip)	Home	Telerehab app (iPad; Apple Inc.)	✓	✓	✓	✓

a C: coordination.

b A: alignment.

c M: motivation.

d S: strength.

e Not applicable.

f ACL: anterior cruciate ligament.

g AR: augmented reality

h VR: virtual reality.

i mHealth: mobile health.

j IMU: inertial measurement unit.

k TRAK: Taxonomy for the Rehabilitation of Knee Conditions.

The included studies represent a diverse geographical distribution, with significant contributions from Asia (eg, [24,25,31]), Europe (eg, [1,32,38]), and North America (eg, [10,39]). The participant cohorts were predominantly postarthroplasty (total knee arthroplasty [TKA] and total hip arthroplasty [THA]) patients (eg, [15,20,21]), with sample sizes ranging from small-scale pilot feasibility trials to large-scale multicenter randomized controlled trials (n=300, [1]). In terms of technological deployment, wearable inertial measurement units (IMUs) and mobile health (mHealth) applications were the most frequently used modalities, reflecting a clinical priority for portable and scalable solutions.

The temporal distribution of included studies shows a gradual increase in publication volume across the review period (Figures 3 and 4): 5 studies [1,39-42] in 2020, 1 in 2021, 7 in 2022, 11 in 2023, 5 in 2024, and 6 in 2025, with 2023‐2025 collectively accounting for 63% (22/35) of the corpus. This pattern suggests growing research activity in the postpandemic period, though the limited number of studies per year precludes formal trend analysis.

**Figure 3.**
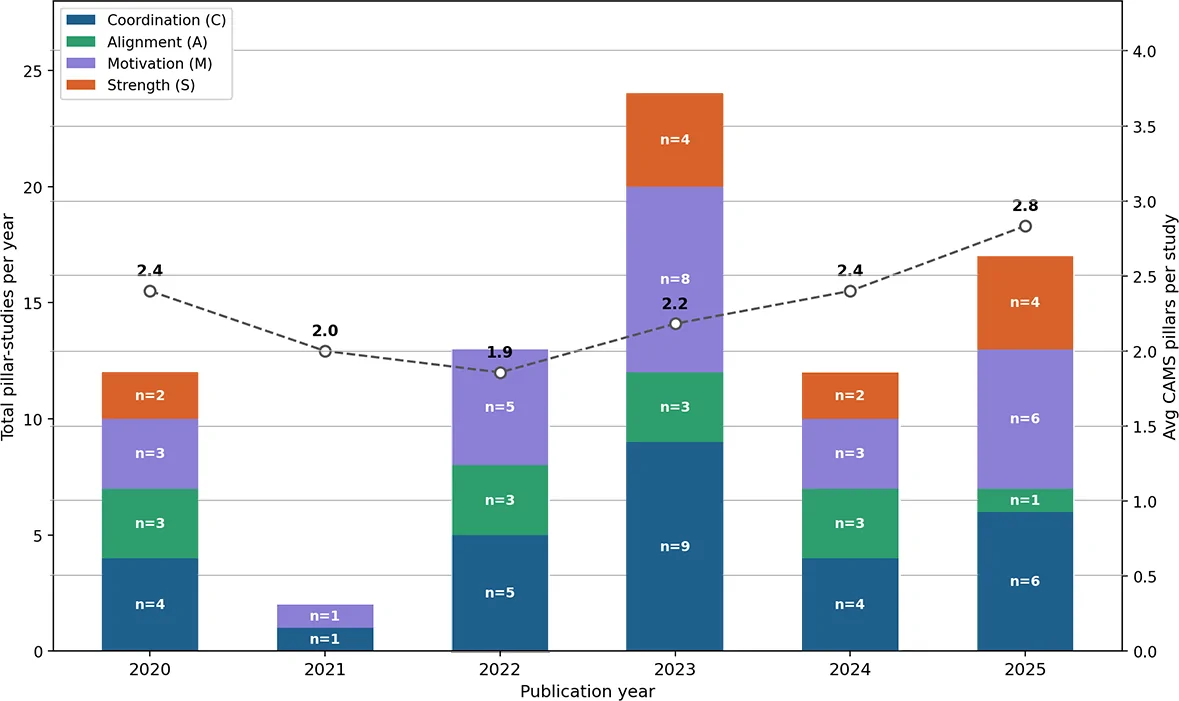
Longitudinal evolution and thematic density of Coordination-Alignment-Motivation-Strength (CAMS) pillars among 35 studies [1,3,10-42] of digital postsurgical orthopedic rehabilitation technologies published between 2020 and 2025. Avg: average.

**Figure 4.**
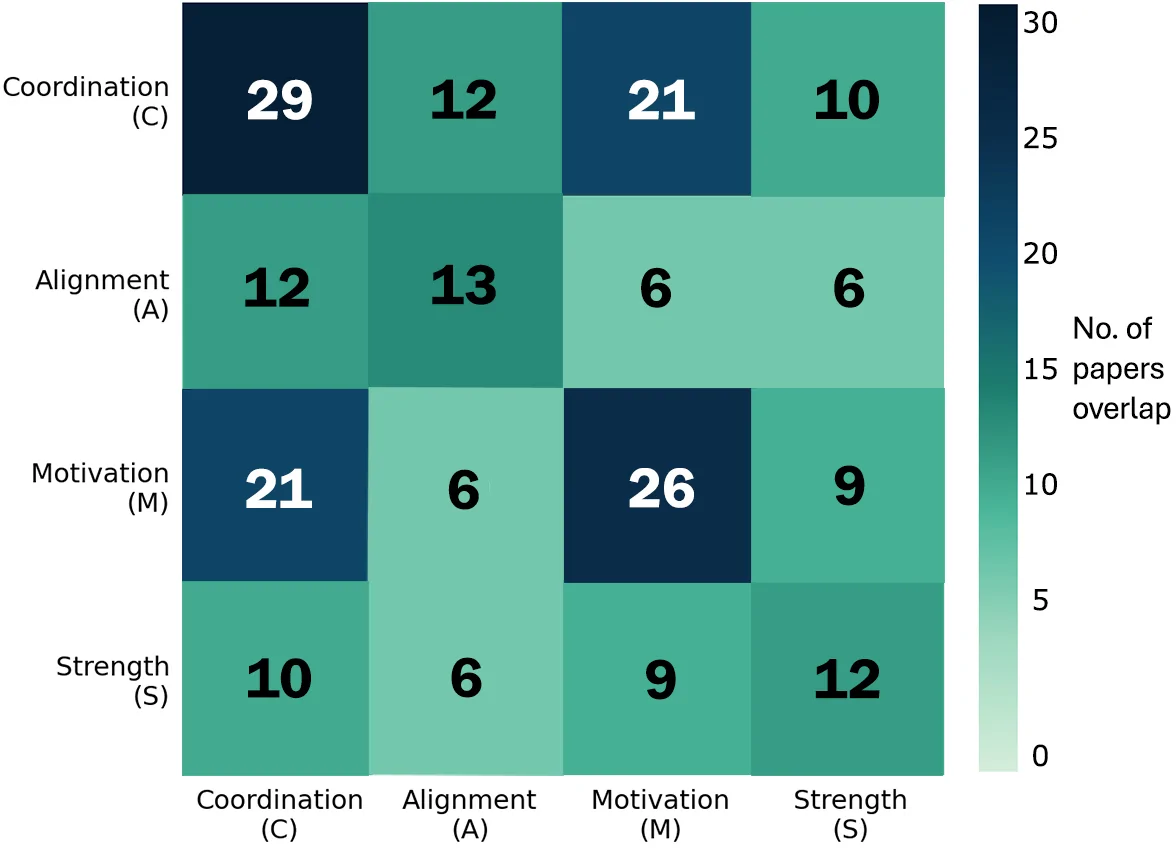
Heatmap of synergy and overlap between Coordination-Alignment-Motivation-Strength (CAMS) pillars among 35 studies [1,3,10-42] of digital postsurgical orthopedic rehabilitation technologies published between 2020 and 2025.

### Methodological Quality and RoB

The methodological profile of the included studies was characterized using design-appropriate appraisal tools (RoB 2 for RCTs, NOS for observational studies, MINORS for nonrandomized designs). As detailed in Table 2, 49% (17/35) of the studies were rated low risk overall on their respective primary appraisal tool, 46% (16/35) demonstrated some concerns, and 6% (2/35) were rated high risk. The temporal distribution of RoB 2 ratings (Table 3) shows that studies published between 2024 and 2025 (eg, [12,16]) more commonly achieved low risk overall ratings compared to earlier pilot studies, though the small number of studies per year means this observation is descriptive rather than inferential.

**Table 2.** Methodological quality appraisal of 35 studies [1,3,10-42] of digital postsurgical orthopedic rehabilitation technologies published between 2020 and 2025, assessed using the Cochrane risk of bias 2 tool (n=32 randomized trials) [1,3,10-22,24-30,32-38,40-42], the Newcastle-Ottawa Scale (n=2 observational studies) [23,31], and the Methodological Index for Non-Randomized Studies (n=1 nonrandomized designs) [39].

No.	Reference no.	Design	Tool	D1^a^	D2^b^	D3^c^	D4^d^	D5^e^	Overall	PEDro^f^ score	Key methodological characteristic
1	Chen et al [10]	RCT^g^	RoB2^h^	L^i^	L	L	L	L	L	8	—^j^
2	Dajpratham et al [11]	RCT	RoB2	L	L	L	L	L	L	8	—
3	O’Reilly et al [12]	RCT	RoB2	L	L	L	L	L	L	8	—
4	Sadiq et al [13]	RCT	RoB2	L	S^k^	L	L	L	L	9	Lack of participant or therapist blinding
5	Yuan et al [14]	RCT	RoB2	L	L	L	L	L	L	8	—
6	Zhou et al [15]	RCT	RoB2	L	S	L	S	L	S	7	Reliance on subjective PROMs^l^ (HHS^m^)
7	Christiansen et al [16]	RCT	RoB2	L	L	L	L	L	L	8	—
8	Lim et al [3]	RCT	RoB2	L	L	L	L	L	L	9	—
9	Xie et al [17]	RCT	RoB2	L	S	L	L	L	L	7	Inherent lack of participant or therapist blinding
10	Zhao et al [18]	RCT	RoB2	S	S	S	S	L	S	6	Unclear allocation concealment and pilot sample size
11	Shin and Hong [19]	RCT	RoB2	S	L	L	L	L	S	—	Randomization sequence detail not specified
12	Guo et al [20]	RCT	RoB2	L	L	L	L	L	L	8	High attrition rate in intervention group
13	Duong et al [21]	RCT	RoB2	L	L	L	L	L	L	8	—
14	Höher et al [22]	RCT	RoB2	L	S	L	S	L	S	6	Monitoring focus with subjective outcomes
15	Lebleu et al [23]	Cohort	NOS	—	—	—	—	—	L	8	Multicenter cohort; no randomization
16	Osterloh et al [24]	RCT	RoB2	L	L	L	S	L	L	7	Use of self-reported functional outcome
17	Shim et al [25]	RCT	RoB2	L	L	L	L	L	L	8	—
18	Shim et al [26]	RCT	RoB2	S	S	S	L	S	S	—	Abstract-only evidence; domains largely unclear
19	Gsangaya et al [27]	RCT	RoB2	S	L	L	S	L	S	—	Unblinded investigators and subjects for subjective outcomes
20	Gür et al [28]	RCT	RoB2	L	H^n^	S	S	H	H	—	Confirmed selective reporting concern (independent SR^o^ verification)
21	Nuevo et al [29]	RCT	RoB2	L	S	L	L	L	S	—	Unblinded treating clinicians with dashboard access
22	Mojica et al [30]	RCT	RoB2	S	S	S	S	L	S	—	Randomization or blinding detail limited
23	Hong et al [31]	Retrospective	NOS^p^	—	—	—	—	—	S	7	Reliance on patient-reported WOMAC^q^ scores
24	Marley et al [32]	RCT	RoB2	L	S	L	S	L	S	7	Detection bias in gamified feedback delivery
25	Pournajaf et al [33]	RCT	RoB2	S	L	S	L	L	S	—	Randomization sequence or attrition detail limited
26	Fuchs et al [34]	RCT	RoB2	H	S	S	S	L	H	—	Quasi-random (time-based) allocation; unblinded subjective outcomes
27	Zavala-González et al [35]	RCT	RoB2	L	L	L	L	L	L	—	—
28	Fascio et al [36]	RCT	RoB2	S	S	S	S	S	S	—	Abstract-only evidence; domains largely unclear
29	Şahi̇n et al [37]	RCT	RoB2	S	S	S	S	L	S	—	Abstract-only evidence; domains largely unclear
30	Dunphy et al [38]	Feasibility RCT	RoB2	L	L	L	L	L	L	8	—
31	Prvu Bettger et al [1]	RCT	RoB2	L	L	L	L	L	L	8	—
32	Wijnen et al [39]	Non-RCT	MINORS^r^	—	—	—	—	—	S	7	Nonrandomized; historical controls
33	Bell et al [40]	RCT	RoB2	L	S	S	S	L	S	—	Unblinded outcome assessor; substantial prebaseline attrition
34	Gianola et al [41]	RCT	RoB2	L	L	L	L	L	L	—	—
35	Nelson et al [42]	RCT	RoB2	S	L	S	L	L	S	—	Allocation concealment method not specified

a D1: randomization process.

b D2: deviations from intended interventions.

c D3: missing outcome data.

d D4: measurement of the outcome.

e D5: selection of the reported result.

f PEDro: Physiotherapy Evidence Database.

g RCT: randomized controlled trial.

h RoB2: risk of bias 2 tool.

i L: low risk.

j Nonapplicable domain (Newcastle-Ottawa Scale or Methodological Index for Non-Randomized Studies tools do not use Risk of Bias 2 domain structure) or Physiotherapy Evidence Database score not independently verified.

k S: some concerns.

l PROM: patient-reported outcome measure.

m HHS: Harris Hip Score.

n H: high risk.

o SR: systematic review.

p NOS: Newcastle-Ottawa Scale.

q WOMAC: Western Ontario and McMaster Universities Osteoarthritis Index.

r MINORS: Methodological Index for Non-Randomized Studies.

**Table 3.** Longitudinal evolution of methodological rigor among 35 [1,3,10-42] studies of digital postsurgical orthopedic rehabilitation technologies published between 2020 and 2025, based on overall risk of bias ratings by publication year.

Year	Low risk, n (%)	Some concerns, n (%)	High risk, n (%)	Total studies, n
2020	2 (40)	3 (60)	0 (0)	5
2021	1 (100)	0 (0)	0 (0)	1
2022	1 (14)	5 (71)	1 (14)	7
2023	5 (45)	5 (45)	1 (9)	11
2024	3 (60)	2 (40)	0 (0)	5
2025	5 (83)	1 (17)	0 (0)	6
Total	17(49)	16 (46)	2 (6)	35

Domain-based analysis (Figure 5) shows that selection and reporting biases were minimal; albeit performance bias (Domain 2) is an intrinsic issue with digital health trials because blinding participants and the therapists is technically impossible (eg, [32]). However, the large mean of PEDro (7.7) indicates that there is a serious focus on objective outcome measurement and high-statistic reporting throughout the corpus. It is important to note that most of the “some concern” ratings were signaled by the use of patient-reported outcome measures (PROMs) in unblinded designs, as opposed to inherent weaknesses in randomization or data integrity.

**Figure 5.**
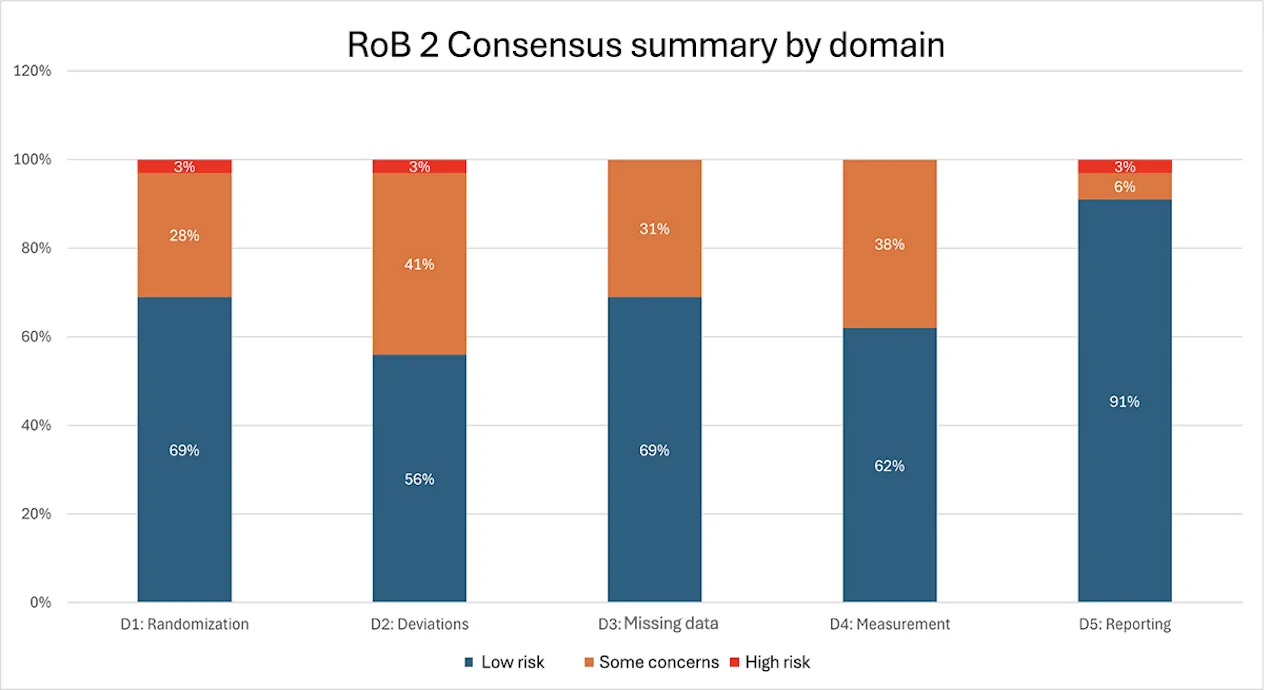
Risk of bias 2 domain-level ratings among the 32 randomized controlled trials [1,3,10-22,24-30,32-38,40-42] of digital postsurgical orthopedic rehabilitation technologies; the remaining 3 nonrandomized studies were appraised using different tools with noncomparable domain structures (Table 2). RoB2: Risk of Bias 2

Each included study contributed an independent patient cohort—no studies shared participants. Patient cohorts were predominantly postarthroplasty (total knee or hip arthroplasty; n=26 studies; Table 1) or post-ACL reconstruction (n=5 studies), with the remaining studies covering rotator cuff repair, meniscectomy, hip fracture, or mixed lower limb populations (n=4 studies). The range of digital interventions was broad: wearable sensor or IMU systems and VR or AR platforms were most common (n=10 each), followed by mHealth applications (n=9) and other telerehabilitation platforms (n=6). Several studies used multimodal combinations of these modalities. Rehabilitation duration across studies ranged from single inpatient sessions (2‐4 days postoperatively) to 24 weeks, with home- and hybrid-based interventions spanning a wide range of postoperative timeframes, from brief early interventions to extended multiweek programs. Intervention dosing varied considerably—from predominantly unsupervised home sessions (eg, [3,15]) to more clinician-supported telehealth models (eg, [11,20])—reflecting the absence of standardized digital rehabilitation protocols in this literature. These characteristics are summarized in Table 1.

### The Lower Limb Dominance: Biomechanical and Economic Drivers

The anatomy of the covered studies focused on the lower limb (32/35, 91%) and targeted the recovery interventions for total knee and hip arthroplasty. This distribution may reflect 2 convergent factors, though causal inference is beyond the scope of this review. First, from a biomechanical perspective, lower limb rehabilitation often prioritizes repetitive, sagittal-plane movements (eg, knee flexion or extension) and gait metrics, which are more easily quantified by current IMUs and markerless vision systems than the complex, multiplanar kinematics of the upper limb (eg, shoulder circumduction). Second, health economic pressures play a significant role; the high volume of lower limb arthroplasty procedures globally has created an urgent clinical need for scalable, automated solutions to reduce hospital length of stay and postoperative physiotherapy costs.

### The Shift Toward Decentralized Care: From Supervision to Autonomy

Regarding the clinical delivery environment, a decisive shift from hospital-centric to decentralized care is evident. The hybrid model was the most frequently used approach (14/35, 40%). This model reflects a “safety-first” innovation strategy, where initial in-clinic supervision ensures patient safety and device calibration, followed by a transitioned digital home program to promote long-term autonomy.

The emergence of protocols that are purely home-based and remote (13/35, 37%) is the second wave of digital orthopedics, which can be enabled through the enhancements in user interface design and the reliability of remote monitoring. This pattern coincides with the accelerated adoption of telerehabilitation during the COVID-19 pandemic, during which remote-only pathways demonstrated clinical feasibility across multiple orthopedic contexts [31,38]. On the other hand, 8 studies used an exclusively clinical delivery model (8/35, 23%), suggesting that digital tools are increasingly deployed to extend rather than replace in-person care but as force multipliers that can be deployed in the domestic setting of the patient to close the critical continuity-of-care gap after discharge.

### Technological Modalities and System Architecture

The technological modalities analysis (Figure 6) indicates that there is a hierarchical deployment of hardware. Wearable IMUs are widely used for objective monitoring and are used in the majority of studies of the lower limbs to measure the angle of joints and gait parameters. Nonetheless, one of the major trends is the use of these sensors with mHealth ecosystems.

**Figure 6.**
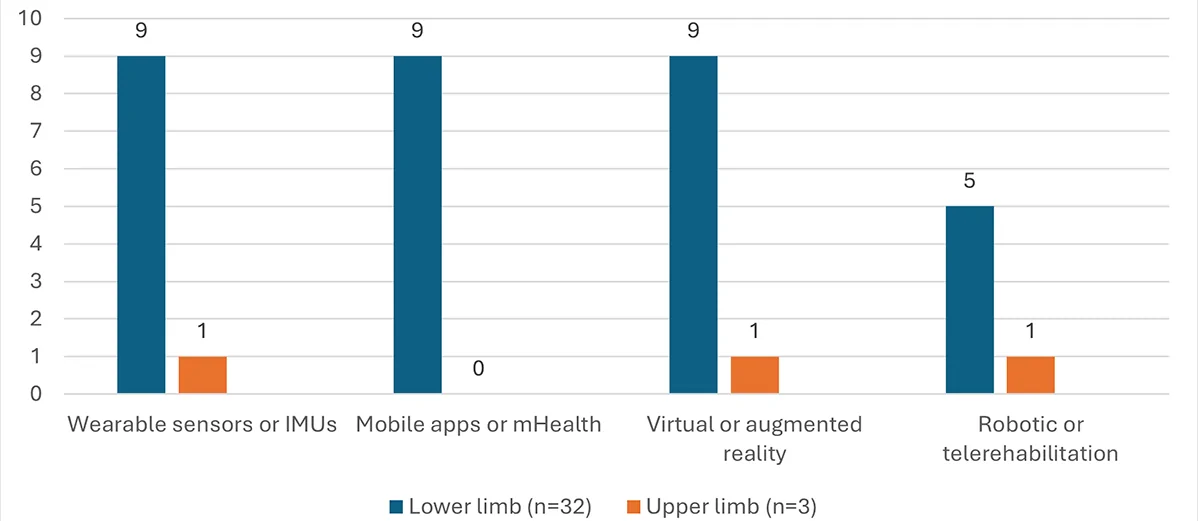
Distribution of technological modalities across anatomical targets among 35 studies of digital postsurgical orthopedic rehabilitation technologies published between 2020 and 2025. IMU: inertial measurement unit; mHealth: mobile health.

The most innovative change that was observed in the collected studies, for the time frame of 2024‐2025, is the emergence of markerless computer vision and AR. These systems (eg, [3]) can reduce the patient’s physical load, that is, by substituting physical sensors with camera-based pose estimation. The more recent studies (2023‐2025) showed increasing architectural diversity, ranging from cloud-based asynchronous monitoring platforms to immersive VR or AR systems and synchronous telehealth models, reflecting the field’s ongoing exploration of different digital delivery paradigms rather than convergence on a single dominant architecture.

### Longitudinal Evolution of the CAMS Pillars

Figure 3 illustrates the distribution of CAMS pillars addressed by publication year. Between 2020 and 2022, when annual study counts ranged from 1 to 7 per year, interventions most frequently addressed Coordination (10/13, 77%) and Motivation (9/13, 69%), while Alignment (6/13, 46%) and, in particular, Strength (2/13, 15%) were less commonly addressed. Between 2023 and 2025, Coordination (19/22, 86%), Motivation (17/22, 77%), and Strength (10/22, 45%) coding were more frequent than in the earlier period, whereas Alignment coding was less frequent (7/22, 32%); however, given the small number of studies per year, these patterns are reported descriptively and should not be interpreted as a defined developmental sequence. The average number of CAMS pillars addressed per study ranged from 1.9 (SD 0.7) in 2022 to 2.8 (SD 0.8) in 2025, with no clear monotonic trend across the review period (Figure 3). This variability reflects heterogeneity in study scope and intervention type, though the data are consistent with a modest shift toward more multidimensional digital interventions in recent years.

### Thematic Synthesis of CAMS Pillars

This section synthesizes the functional outcomes and technological architecture across the n=35 corpus. The results reveal that the current digital landscape is defined by multimodal integration. As illustrated in the synergy heatmap (Figure 4), Coordination and Motivation were the most frequently coaddressed pillars, appearing together in 60% (21/35) of included studies, suggesting this pairing functions as the functional baseline for current digital rehabilitation tools.

### Motivation: The Digital Anchor

The most widespread pillar (26/35, 74%) was motivation, which became the main factor in influencing longitudinal adherence in the unsupervised domestic environment. The synthesis reveals an evolution from extrinsic motivators (eg, automated SMS reminders and adherence logs) to intrinsic engagement through immersive gamification.

VR and high-engagement digital interventions (eg, [2]) have been associated with reduced pain perception and perceived exertion during physical activity in the included literature, though this review’s descriptive design does not establish a causal or clinical analgesic effect. Leveraging the “distraction effect,” digital interventions may redirect emphasis on the postsurgical mechanical discomfort of movement toward goal-oriented play; these digital health technologies (DHTs) may thereby help address psychological obstacles to rehabilitation. This engagement set the basis of the high-frequency repetition volume needed to stimulate effective tissue remodeling and functional recovery.

### Coordination-Motivation Axis: The Engagement-Precision Loop

Coordination and Motivation are identified as the dominant synergy. The clinical importance of this relationship is high, as high-frequency repetition is an element of the precision-based motor learning (Coordination) that could be retained only with the help of psychological engagement (Motivation). By integrating real-time biofeedback, such as AR-based movement guidance (eg, [3]), with gamified reward structures, DHTs support precise motor execution within an engaging framework. This process may reduce the cognitive load on the patient, transitioning exercise from a monitored chore to a goal-oriented task, which is a prerequisite for achieving the high dose of training required for neural plasticity.

### Alignment as a Gatekeeper for Coordination

While Coordination tracks the path of movement, Alignment is intended to assess the integrity of that path. The 17% (6/35) overlap between these pillars (eg, [3,13,25]) indicates an emerging trend toward “Gait-Aware” systems. Without Alignment, Coordination tracking is at risk of validating “compensatory movements”—where a patient achieves a coordinate goal by using maladaptive postures. AI-driven markerless vision may function as a monitoring tool for postural alignment, flagging compensatory movement patterns before Coordination metrics are recorded; whether this translates to reduced risk of abnormal loading at the surgical site was not assessed by the included studies.

### The “Strength Isolation”: A Technological Bottleneck

According to the heatmap, the Strength pillar had the lowest co-occurrence with Alignment (6/35, 17%), indicating that studies addressing strength rarely also addressed postural alignment. This isolation may point to a technological bottleneck: current digital platforms appear to demonstrate greater capability for monitoring kinematics (tracking the quality of motion) than for kinetic measurement (force quantification). Strength interventions currently function as standalone silos (eg, [24]), lacking the integrated biofeedback seen in the coordination-motivation axis. This gap may also reflect clinical and safety factors, including conservative protocols for early postoperative resistance training and publication trends favoring adherence studies over strength training trials.

## Discussion

### Summary of Principal Findings

Examined through the CAMS framework, the current corpus of digital orthopedic rehabilitation studies reveals a descriptive imbalance between behavioral engagement and physical load assessment. Coordination (29/35, 83%) and Motivation (26/35, 74%) were the most commonly addressed domains, reflecting the maturation of engagement-focused and movement-tracking technologies in home-based settings. By contrast, Strength-focused interventions appeared in only 34% (12/35) of studies, most of which required clinically supervised resistance hardware, limiting their applicability in decentralized care models. This gap, between kinematic monitoring (tracking the quality of movement) and kinetic measurement (quantifying mechanical force), is the central finding of this review.

The shift toward hybrid (14/35, 40%) and home-based (13/35, 37%) delivery models across the corpus is consistent with the broader adoption of telerehabilitation during and after the COVID-19 pandemic. This distributional pattern suggests that decentralized care has moved from being a contingency measure to an established component of orthopedic rehabilitation pathways, though the heterogeneity of included interventions and outcome measures means these observations are descriptive rather than conclusive.

### From Kinetic Monitoring to Kinetic Integration

The co-occurrence of 60% (21/35) between Coordination and Motivation pillars (Figure 4) suggests that this pairing forms the functional core of current digital rehabilitation tools. Conventional physiotherapy relies on direct clinician observation for corrective feedback; current AR-based and IMU-based systems (eg, [3]) replicate elements of this closed-loop feedback in nonsupervised home settings, supporting the neuromuscular repetition volumes required for motor relearning.

### Clinical Implications

The prevalence of hybrid delivery (14/35, 40%) suggests that a phased model, initial in-clinic assessment followed by digital home-based maintenance, may represent an emerging standard of care for postsurgical orthopedic rehabilitation. This model balances safety and therapist oversight in the acute phase with the scalability and patient autonomy of digital tools during extended recovery. The growing availability of markerless AI-based alignment monitoring [3,13] further supports this model, removing the need for sensor placement and enabling higher-quality alignment tracking for patients with limited technological literacy.

Based on the evidence mapped in this review, the following principles are suggested for clinical consideration. Platforms addressing the Coordination-Motivation axis alongside Alignment offer broader functional coverage and may be preferable to single-pillar adherence tools. Markerless vision systems should be considered for early detection of compensatory movement patterns in home settings. Clinicians should recognize that current DHTs are predominantly kinematic — direct strength measurement requires supplementary in-clinic assessment until affordable kinetic wearables become available. Hybrid delivery models offer a pragmatic balance between clinical oversight and home-based scalability. These suggestions are based on descriptive mapping findings and require validation through comparative clinical trials.

### Limitation and Future Directions

This review has several limitations. First, the CAMS framework is a novel, unvalidated taxonomy proposed here as an exploratory classification tool; its categories reflect the authors’ conceptual mapping rather than empirically derived dimensions. Second, the high degree of heterogeneity across intervention types, outcome measures, dosing protocols, and follow-up periods precluded formal meta-analysis, and the descriptive findings cannot be interpreted causally. Third, performance bias is an inherent challenge in digital health trials where blinding participants and therapists is not feasible, and most included studies relied at least partially on patient-reported outcomes. Fourth, Web of Science was not searched due to lack of institutional access, which may have resulted in missed records not indexed in the 5 databases used, though substantial overlap with Scopus and IEEE Xplore is expected for this multidisciplinary technology-focused literature. Finally, intervention durations varied considerably across studies, ranging from single inpatient sessions to programs extending several months postsurgery, leaving long-term functional durability, including joint longevity and secondary injury prevention, largely unaddressed in the literature, regardless of intervention length.

Future research should prioritize 3 directions. First, empirical validation of the CAMS framework across diverse clinical and technological settings is needed to establish its utility as a classification and prescriptive tool. Second, trials specifically targeting the Strength pillar, particularly studies integrating smart resistance hardware with kinematic monitoring systems, are required to close the gap identified in this review. Third, longitudinal studies with follow-up extending well beyond the early postoperative period are needed to evaluate whether digital rehabilitation pathways support durable functional recovery and safe return to sport.

### Conclusions

This scoping review mapped 35 [1,3,10-42] studies of digital technologies used in postsurgical orthopedic rehabilitation between 2020 and 2025, using the proposed CAMS framework to characterize their functional coverage. Within this literature, Coordination and Motivation were the most frequently addressed domains, while Alignment and, in particular, Strength were less consistently represented. This pattern may reflect current technological capabilities for quantifying postural correction and mechanical load, though the descriptive nature of this review precludes definitive conclusions about the underlying causes. Hybrid delivery models, combining initial clinical supervision with subsequent home-based digital support, were the most common approach across the included studies. These findings suggest several directions for future research, including empirical validation of the CAMS framework, development of accessible tools for quantifying physiological load, and longitudinal studies evaluating functional outcomes beyond the early postoperative period. As an exploratory taxonomy, CAMS may offer a useful starting point for classifying and comparing digital rehabilitation technologies in future research.

## Supplementary material

10.2196/100548 Checklist 1 PRISMA-ScR checklist.

## Abbreviations

ACL: anterior cruciate ligament
AR: augmented reality
CAMS: Coordination, Alignment, Motivation, and Strength
CENTRAL: Cochrane Central Register of Controlled Trials
DHT: digital health technology
IMU: inertial measurement unit
mHealth: mobile health
MINORS: Methodological Index for Non-Randomized Studies
NOS: Newcastle-Ottawa Scale
PCC: population-concept-context
PEDro: Physiotherapy Evidence Database
PRISMA-ScR: Preferred Reporting Items for Systematic Reviews and Meta-Analyses extension for Scoping Reviews
PROM: patient-reported outcome measure
PROSPERO: International Prospective Register of Systematic Reviews
RCT: randomized controlled trial
RoB2: Cochrane risk-of-bias tool, version 2, for randomized trials
THA: total hip arthroplasty
TKA: total knee arthroplasty
TRAK: Taxonomy for the Rehabilitation of Knee Conditions
VR: virtual reality

## References

[1] Prvu Bettger J, Green CL, Holmes DN (2020). Effects of virtual exercise rehabilitation in-home therapy compared with traditional care after total knee arthroplasty: VERITAS, a randomized controlled trial. J Bone Joint Surg Am.

[2] Zitti M, Regazzetti M, Federico S (2025). Effectiveness of virtual reality for pain management in musculoskeletal disorders across anatomical regions: a systematic review and meta-analysis. Musculoskeletal Care.

[3] Lim JY, Yu HJ, Kim SH (2024). Effectiveness of in-home, augmented reality-based telerehabilitation after anterior cruciate ligament reconstruction: a randomized controlled trial. Orthop J Sports Med.

[4] Kim N (2025). Emerging role of visual biofeedback in rehabilitation following ACL reconstruction. Asian J Kinesiol.

[5] Hu R, Diao Y, Wang Y (2023). Effective evaluation of HGcnMLP method for markerless 3D pose estimation of musculoskeletal diseases patients based on smartphone monocular video. Front Bioeng Biotechnol.

[6] Seo NJ, Coupland K, Finetto C, Scronce G (2024). Wearable sensor to monitor quality of upper limb task practice for stroke survivors at home. Sensors (Basel).

[7] Khalid S, Alnajjar F, Gochoo M, Renawi A, Shimoda S (2023). Robotic assistive and rehabilitation devices leading to motor recovery in upper limb: a systematic review. Disabil Rehabil Assist Technol.

[8] Aprile IG, Quaglini S, Turchetti G (2025). Rehabilitation robotics and allied digital technologies: opportunities, barriers and solutions for improving their clinical implementation. A position paper from the Fit for Medical Robotics Initiative. Front Robot AI.

[9] Tricco AC, Lillie E, Zarin W (2018). PRISMA Extension for Scoping Reviews (PRISMA-ScR): Checklist and Explanation. Ann Intern Med.

[10] Chen W, Qin X, Yang H, Tan Z, Liu P (2025). Impact of cloud-based follow-up exercise prescription on knee function and quality of life in homebound patients post-total knee arthroplasty: a randomized controlled trial. J Orthop Surg Res.

[11] Dajpratham P, Komas J, Yamthed R (2025). Feasibility and efficacy of real-time teleresistance exercise programs for physical function in elderly patients after hip fracture surgery: a randomized controlled trial. BMC Geriatr.

[12] O’Reilly OC, Bozoghlian M, Glass N (2025). Physical therapy following shoulder arthroplasty: an assessment of telerehabilitation vs. in-person physical therapy. Semin Arthroplasty JSES.

[13] Sadiq S, Noor R, Akram R (2025). Effect of lifestyle modification through web-based telerehabilitation monitoring combined with supervised sensorimotor training after total knee arthroplasty: randomized controlled trial. JMIR Mhealth Uhealth.

[14] Yuan C, Xu C, Huang L, He Y, Lu S (2025). The efficacy and cost-effectiveness analysis of telerehabilitation for patients after arthroscopic ACL-reconstruction: a non-inferiority randomized controlled trial. J Orthop Surg Res.

[15] Zhou Y, Lyu Y, Wang Q, Ma Y, Huang L, Zhang X (2024). Mobile-based in-home telerehabilitation compared with in-hospital face-to-face rehabilitation for elderly patients after total hip arthroplasty in China’s level 1 trauma center: a noninferiority randomized controlled trial. Front Surg.

[16] Christiansen CL, Kline PW, Anderson CB (2024). Optimizing total knee arthroplasty rehabilitation with telehealth physical activity behavior change intervention: a randomized clinical trial. Phys Ther.

[17] Xie W, He M, Zheng S (2024). Clinical application research of intelligent monitoring system for knee rehabilitation: a randomized controlled trial. J Orthop Surg Res.

[18] Zhao R, Cheng L, Zheng Q (2024). A smartphone application-based remote rehabilitation system for post-total knee arthroplasty rehabilitation: a randomized controlled trial. J Arthroplasty.

[19] Shin D, Hong S (2024). A study on the effectiveness of VR rehabilitation training content for older individuals with total knee replacement: pilot study. Health Care (Don Mills).

[20] Guo Y, Li D, Wu Y bo, Sun X, Sun X ying, Yang Y ping (2023). Mobile health-based home rehabilitation education improving early outcomes after anterior cruciate ligament reconstruction: a randomized controlled clinical trial. Front Public Health.

[21] Duong V, Robbins SR, Dennis S, Venkatesha V, Ferreira ML, Hunter DJ (2023). Combined digital interventions for pain reduction in patients undergoing knee replacement: a randomized clinical trial. JAMA Netw Open.

[22] Höher J, Lischke B, Petersen W (2023). Sensor-based telerehabilitation system increases patient adherence after knee surgery. PLOS Digit Health.

[23] Lebleu J, Pauwels A, Anract P, Parratte S, Van Overschelde P, Van Onsem S (2023). Digital rehabilitation after knee arthroplasty: a multi-center prospective longitudinal cohort study. J Pers Med.

[24] Osterloh J, Knaack F, Bader R (2023). The effect of a digital-assisted group rehabilitation on clinical and functional outcomes after total hip and knee arthroplasty-a prospective randomized controlled pilot study. BMC Musculoskelet Disord.

[25] Shim GY, Kim EH, Baek YJ (2023). A randomized controlled trial of postoperative rehabilitation using digital healthcare system after rotator cuff repair. NPJ Digit Med.

[26] Shim GY, Kim EH, Lee SJ (2023). Postoperative rehabilitation using a digital healthcare system in patients with total knee arthroplasty: a randomized controlled trial. Arch Orthop Trauma Surg.

[27] Gsangaya MR, Htwe O, Selvi Naicker A (2023). Comparison between the effect of immersive virtual reality training versus conventional rehabilitation on limb loading and functional outcomes in patients after anterior cruciate ligament reconstruction: a prospective randomized controlled trial. Asia Pac J Sports Med Arthrosc Rehabil Technol.

[28] Gür O, Başar S (2023). The effect of virtual reality on pain, kinesiophobia and function in total knee arthroplasty patients: a randomized controlled trial. Knee.

[29] Nuevo M, Rodríguez-Rodríguez D, Jauregui R (2024). Telerehabilitation following fast-track total knee arthroplasty is effective and safe: a randomized controlled trial with the ReHub® platform. Disabil Rehabil.

[30] Mojica ES, Vasavada K, Hurley ET (2023). Despite equivalent clinical outcomes, patients report Less satisfaction with telerehabilitation versus standard in-office rehabilitation after arthroscopic meniscectomy: a randomized controlled trial. Arthrosc Sports Med Rehabil.

[31] Hong M, Loeb J, Yang M, Bailey JF (2022). Postoperative outcomes of a digital rehabilitation program after Total knee arthroplasty: retrospective, observational feasibility study. JMIR Form Res.

[32] Marley WD, Barratt A, Pigott T, Granat M, Wilson JD, Roy B (2022). A multicenter randomized controlled trial comparing gamification with remote monitoring against standard rehabilitation for patients after arthroscopic shoulder surgery. J Shoulder Elbow Surg.

[33] Pournajaf S, Goffredo M, Pellicciari L (2022). Effect of balance training using virtual reality-based serious games in individuals with total knee replacement: a randomized controlled trial. Ann Phys Rehabil Med.

[34] Fuchs L, Kluska A, Novak D, Kosashvili Y (2022). The influence of early virtual reality intervention on pain, anxiety, and function following primary total knee arthroplasty. Complement Ther Clin Pract.

[35] Zavala-González J, Martínez D, Gutiérrez-Espinoza H (2022). Effectiveness of adding virtual reality to physiotherapeutic treatment in patients with total hip arthroplasty. A randomized controlled trial. Clin Rehabil.

[36] Fascio E, Vitale JA, Sirtori P, Peretti G, Banfi G, Mangiavini L (2022). Early virtual-reality-based home rehabilitation after total hip arthroplasty: a randomized controlled trial. J Clin Med.

[37] Şahi̇n A, Agar A, Ertürk C (2022). The effect of telerehabilitation on early outcomes in patients undergoing primary total knee replacement: a prospective randomized study. J Surg Med.

[38] Dunphy E, Button K, Hamilton F, Williams J, Spasic I, Murray E (2021). Feasibility randomised controlled trial comparing TRAK-ACL digital rehabilitation intervention plus treatment as usual versus treatment as usual for patients following anterior cruciate ligament reconstruction. BMJ Open Sport Exerc Med.

[39] Wijnen A, Hoogland J, Munsterman T (2020). Effectiveness of a home-based rehabilitation program after total hip arthroplasty driven by a tablet app and remote coaching: nonrandomized controlled trial combining a single-arm intervention cohort with historical controls. JMIR Rehabil Assist Technol.

[40] Bell KM, Onyeukwu C, Smith CN (2020). A portable system for remote rehabilitation following a total knee replacement: a pilot randomized controlled clinical study. Sensors (Basel).

[41] Gianola S, Stucovitz E, Castellini G (2020). Effects of early virtual reality-based rehabilitation in patients with total knee arthroplasty: a randomized controlled trial. Medicine (Baltimore).

[42] Nelson M, Bourke M, Crossley K, Russell T (2020). Telerehabilitation is non-inferior to usual care following total hip replacement - a randomized controlled non-inferiority trial. Physiotherapy.

[43] Feinstein AR, Cicchetti DV (1990). High agreement but low kappa: I. The problems of two paradoxes. J Clin Epidemiol.

[44] Hestetun-Mandrup AM, Toh ZA, Oh HX, He HG, Martinsen ACT, Pikkarainen M (2024). Effectiveness of digital home rehabilitation and supervision for stroke survivors: a systematic review and meta-analysis. Digit Health.

[45] Abedi A, Colella TJF, Pakosh M, Khan SS (2024). Artificial intelligence-driven virtual rehabilitation for people living in the community: a scoping review. NPJ Digit Med.

